# Enriched Music-supported Therapy for chronic stroke patients: a study protocol of a randomised controlled trial

**DOI:** 10.1186/s12883-020-02019-1

**Published:** 2021-01-12

**Authors:** Jennifer Grau-Sánchez, Emma Segura, David Sanchez-Pinsach, Preeti Raghavan, Thomas F. Münte, Anna Marie Palumbo, Alan Turry, Esther Duarte, Teppo Särkämö, Jesus Cerquides, Josep Lluis Arcos, Antoni Rodríguez-Fornells

**Affiliations:** 1 Cognition and Brain Plasticity Unit, Bellvitge Biomedical Research Institute, L’Hospitalet de Llobregat, 08907 Barcelona, Spain; 2 Escola Universitària d’Infermeria i Teràpia Ocupacional de Terrassa, Autonomous University of Barcelona, C/ de la Riba, 90, 08221 Terrassa, Spain; 3 Institute of Neurosciences, University of Barcelona, 08035 Barcelona, Spain; 4 Artificial Intelligence Research Institute, Spanish National Research Council, Bellaterra, 08193 Barcelona, Spain; 5 Department of Physical Medicine and Rehabilitation, John Hopkins University, Baltimore, MD 21287 USA; 6 Department of Neurology, University of Lübeck, 23562 Lübeck, Germany; 7 Nordoff-Robbins Center for Music Therapy, New York University, New York, 10012 USA; 8 Rehabilitation Science Program, Steinhardt School of Culture, Education and Human Development, New York University, 10003 New York, USA; 9 Department of Physical and Rehabilitation Medicine, Hospitals del Mar i l’Esperança, 08003 Barcelona, Spain; 10 Cognitive Brain Research Unit, Department of Psychology and Logopedics, University of Helsinki, 00014 Helsinki, Finland; 11 Department of Cognition, Development and Educational Psychology, University of Barcelona, 08035 Barcelona, Spain; 12 Institució Catalana de Recerca i Estudis Avançats, 08010 Barcelona, Spain

**Keywords:** Stroke, Rehabilitation, Music therapy, Music-supported therapy

## Abstract

**Background:**

Residual motor deficits of the upper limb in patients with chronic stroke are common and have a negative impact on autonomy, participation and quality of life. Music-Supported Therapy (MST) is an effective intervention to enhance motor and cognitive function, emotional well-being and quality of life in chronic stroke patients. We have adapted the original MST training protocol to a home-based intervention, which incorporates increased training intensity and variability, group sessions, and optimisation of learning to promote autonomy and motivation.

**Methods:**

A randomised controlled trial will be conducted to test the effectiveness of this enriched MST (eMST) protocol in improving motor functions, cognition, emotional well-being and quality of life of chronic stroke patients when compared to a program of home-based exercises utilizing the Graded Repetitive Arm Supplementary Program (GRASP). Sixty stroke patients will be recruited and randomly allocated to an eMST group (*n* = 30) or a control GRASP intervention group (*n* = 30). Patients will be evaluated before and after a 10-week intervention, as well as at 3-month follow-up. The primary outcome of the study is the functionality of the paretic upper limb measured with the Action Research Arm Test. Secondary outcomes include other motor and cognitive functions, emotional well-being and quality of life measures as well as self-regulation and self-efficacy outcomes.

**Discussion:**

We hypothesize that patients treated with eMST will show larger improvements in their motor and cognitive functions, emotional well-being and quality of life than patients treated with a home-based GRASP intervention.

**Trial registration:**

The trial has been registered at ClinicalTrials.gov and identified as NCT04507542 on 8 August 2020.

## Background

Stroke is one of the leading causes of long-term disability worldwide [30]. The reduction of mortality rates, especially in developed countries, has resulted in more survivors living with disability and needing rehabilitation and long-term care and support [24, 42]. Many stroke patients experience unilateral paresis of the upper extremity, which affects the individual’s autonomy in basic and instrumental activities of daily living, having a significant impact on participation and quality of life [17, 39, 55, 89].

Rehabilitation programs for stroke patients are usually delivered immediately following the stroke, when the potential for recovery is thought to be the greatest [40, 86]. Rehabilitation aims to enable the individual to achieve the highest possible level of functioning in order to reintegrate the patient into community life [2, 83]. However, once acute rehabilitation ends, the individual affected by stroke faces multiple challenges during the chronic stage. More than one third of individuals live with some residual functional limitations in basic activities, 50% of chronic stroke patients need support with instrumental activities of daily living and 65% suffer restrictions in their reintegration into community life [35, 55]. These residual functional limitations correlate negatively with emotional well-being and life satisfaction, in particular with regard to vocational and leisure activities [37, 38, 91]. Despite the recommendations for maintaining an active lifestyle, stroke survivors show low levels of activity and they may even experience functional deterioration during the chronic phase [6, 12, 31, 58, 92]. However, recovery has been shown to continue well into the chronic stage and depends on dose and intensity of the interventions provided [20]. Several studies have demonstrated that home and community-based interventions can increase functional independence, participation and emotional well-being in chronic stroke patients [13, 29, 34, 78]. In this vein, there is increasing interest in developing and validating interventions for chronic stroke patients aimed at enhancing physical and psychological well-being [12].

One class of music-based intervention for stroke motor rehabilitation involve playing musical instruments with the affected upper extremity following the principles of motor learning and multimodal stimulation [33]. These interventions are feasible to apply in the chronic phase [3, 80]. Among them, Music-Supported Therapy (MST) aims to enhance the motor function of the paretic upper limb by following a standardised program of keyboard and drum exercises [77]. MST includes necessary components for promoting motor learning such as mass repetition of movements, shaping, tailoring, task variability, instructional language and guidance by the therapist, modelling, and feedback [33]. Of particular relevance in MST is audio-motor coupling since individuals receive auditory feedback from the musical instrument that may help detect errors and adjust future movements [73]. Moreover, playing musical instruments is an activity that involves emotional and motivational aspects, which can boost motor learning and enhance emotional well-being [70, 88, 94].

Two recent randomised controlled trials have shown that MST can lead to similar motor improvements as with standard rehabilitation [28, 32]. A study of 20 chronic stroke patients also found that brain plastic changes can occur after MST in the form of cortical motor map reorganisation and enhanced functional coupling between motor and auditory regions [4, 71]. Furthermore, Grau-Sánchez et al. [32] showed that patients with higher sensitivity to music reward were the ones that showed greater motor improvements after MST, a finding that highlights the role of reward and motivation on learning during rehabilitation [1, 32, 63].

Despite these promising findings, the training protocol of MST has some limitations that have precluded widespread application in the chronic stage. First, MST is often delivered in the hospital or in rehabilitation centres; however, this form of delivery may not be the most suitable option for chronic patients. One of the aims of rehabilitation in the chronic phase is reintegration into community life and transitioning to home- and community-based programs can assist with such reintegration [65]. Second, studies investigating MST usually provide between 15 and 20 sessions of 30 min during over three or 4 weeks. This training intensity is not sufficient in the chronic phase, as training protocols need to be of higher intensity and longer duration to effectively promote recovery [20, 40, 41]. Third, only two musical instruments have been used: a keyboard and an electronic drum set, which allows training fine finger movements and gross arm movements. The use of other instruments might improve MST by providing a wider range of movements. Fourth, sessions are provided individually, which does not promote social interaction with other patients and lack a key components of musical activity, social bonding [19, 25, 60]. Especially in the chronic stage, patients with stroke often feel lonely and deprived of opportunities to engage in meaningful social activities [37, 56]. Peer support is particularly relevant in rehabilitation since it can increase patients’ quality of life [44]. Finally, sessions are therapist-led, which in the chronic stage, can diminish the patients’ opportunities to engage in more self-regulated behaviours during their learning process [15]. According to recent theories of motor learning [93], optimization of learning is closely associated with social and intrinsic motivational factors. Therefore, interventions focused on strengthening motivational and self-control aspects could lead to greater recovery [21, 22, 48].

Considering the above constraints and taking into account previous experiences in adapting music interventions for home use [26, 87], we have designed a 10-week enriched MST (eMST) training program combining individual self-training sessions at home with online peer-group sessions. Compared to the standard MST program eMST features i) increased training intensity and range of movements trained; ii) inclusion of peer-group sessions; iii) optimisation of learning through enhanced intrinsic motivational factors to promote more autonomy; and iv) adaptation of the program for home use.

The program comprises 40 one-hour sessions (40 h in total) distributed evenly over 10 weeks (4 sessions/week). We have also included additional percussion instruments to increase the range of movements that patients can train [70]. Considering that participation in social music activities is one of the most important sources of reward derived from music [52], we have included group sessions once a week to promote social interaction and group bonding [59, 82]. The self-training sessions are delivered at home using an electronic tablet, a keyboard and a set of percussion instruments. We have designed an artificial intelligence (AI) platform taking into account recent theories of motor learning to optimise the learning process by boosting intrinsic motivational factors. In the sessions at home, patients are given opportunities to have more control of their behaviours during the training, which can have a significant impact on feelings of self-competence and autonomy. Moreover, the AI platform uses elements of gamification and continuously monitors the patient’s performance and supports therapists in the design and personalization of training sessions by using prediction and prescription components [75]. The adaptation of the program for home use may serve to increase motivation and reward derived from music, facilitate learning through self-controlled training and feedback, and enhance mood and quality of life by improving self-esteem, competence and autonomy.

This parallel-group randomised controlled trial will test the effectiveness of eMST in improving upper extremity motor function in chronic stroke patients when compared to a validated program of home-based exercises. Secondary objectives include testing the effectiveness of this intervention in enhancing cognitive outcomes, emotional well-being, quality of life, and self-regulation and efficacy and compare the effects of this intervention to a conventional home-based program of upper extremity exercises. We hypothesize that eMST will lead to larger motor and cognitive improvements, and enhanced emotional well-being and quality of life compared with home-based physical exercises alone.

## Methods

### Study design

A parallel-group randomised controlled trial will be conducted with participants being randomised to either eMST (eMST-group) or to a control treatment (CT-group), the latter receiving the Graded Repetitive Arm Supplementary Program (GRASP, [36]). The control treatment has already been validated and proven to be effective in enhancing the motor function of the upper extremity in chronic stroke patients [18, 28, 36].

**Fig. 1:**
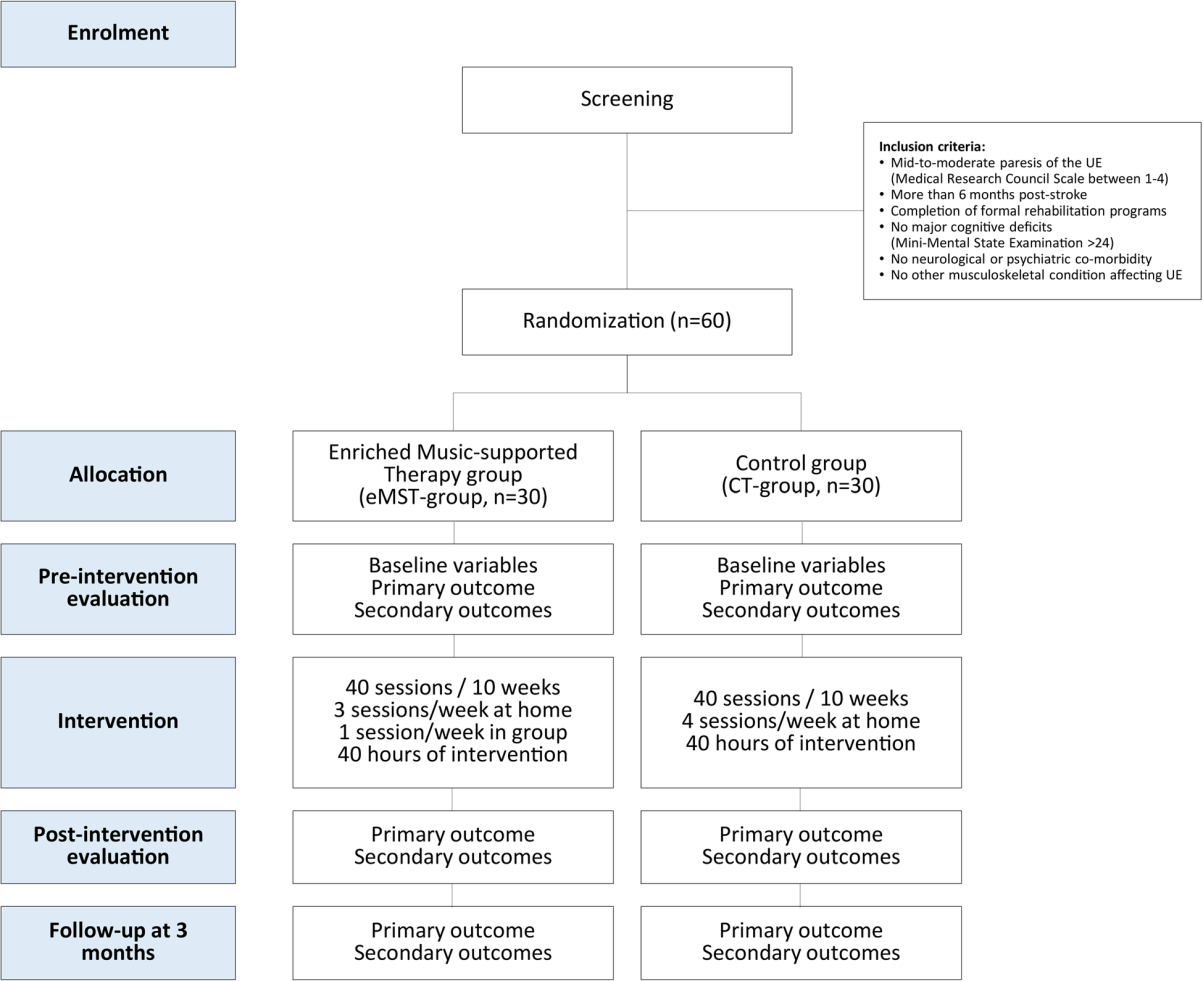
Study design. This study is a parallel-randomised controlled trial with two treatment groups (enriched Music-supported Therapy group [eMST-group, *n* = 30] and Control group [CT-group, *n* = 30]). Participants will be evaluated before and after the intervention as well as in a 3-month follow up

Both interventions will comprise of 40 one-hour sessions distributed over 10 weeks (4 sessions per week, 40 h in total). Before and after the treatment, both groups will undergo an evaluation of their motor and cognitive function, emotional well-being and quality of life. A follow-up assessment will be conducted at 3 months (Fig. 1).

### Participants

Chronic stroke patients will be recruited from two tertiary hospitals of the Barcelona metropolitan area (Hospitals del Mar i l’Esperança and Bellvitge University Hospital). These hospitals have stroke units and specialised inpatient and outpatient neurological rehabilitation departments.

Patients diagnosed with ischemic or haemorrhagic stroke will be eligible as participants if they fulfil the following criteria: presence of mild-to-moderate paresis of the upper extremity after a stroke defined as having a score between 1 and 4 in the Medical Research Council Scale for Muscle Strength at the distal upper limb muscles; more than 6 months post-stroke; completion of formal rehabilitation programs; no major language or cognitive deficits affecting comprehension (Montreal Cognitive Evaluation ≥26); no neurological or psychiatric co-morbidity; and no other musculoskeletal condition affecting upper extremity motor function (e.g. fracture or arthritis).

Two clinical researchers will review medical records of stroke patients who had been treated in the rehabilitation departments of the recruiting hospitals. Phone contacts will be made to screen for potential participants followed by an appointment to evaluate if the patient fulfils the inclusion criteria. Patients will receive a detailed explanation of the procedures of the study and will provide written informed consent prior to participation.

### Experimental treatment: enriched music-supported therapy program

Participants in the eMST-group will follow a 10-week intervention that will consist of 4 weekly one-hour sessions (total program duration: 40 h). The training program comprises three individual home-based self-training sessions and one group session per week.

#### Individual home-based self-training sessions

**Fig. 2:**
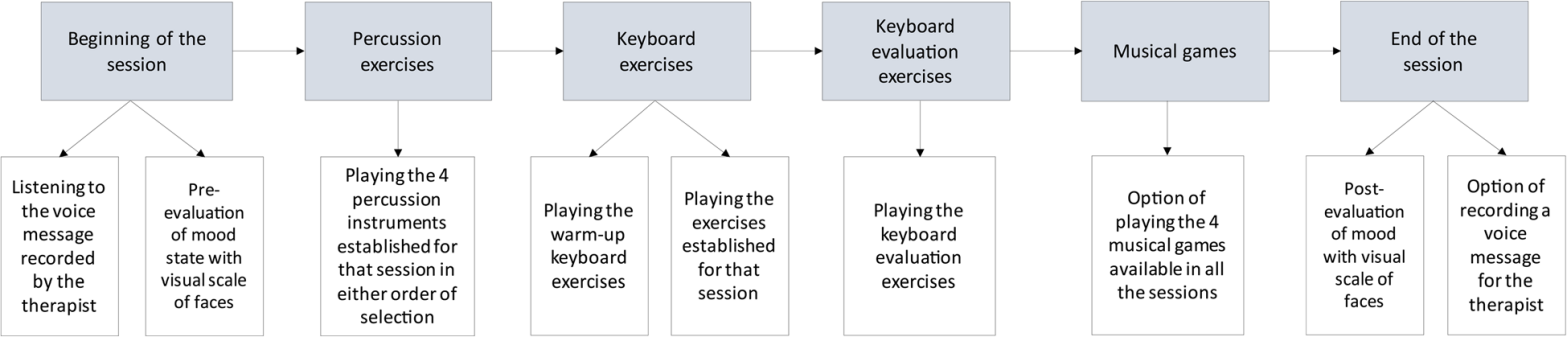
Structure of individual sessions

The individual sessions at home are delivered using an app on electronic tablets, which provides instructions and cues for patients, records the patient’s performance and gathers data about exercises and compliance. At the beginning of the intervention program, a therapist visits the patient’s home to provide the materials for treatment and instructions on the app for home use. The participant is provided with percussion musical instruments (Table 1), an electronic keyboard and an electronic tablet. In each session, the participant is asked to play four percussion instruments and the electronic keyboard to train gross and fine mobility, respectively. Table 1 describes the different movements that can be trained with each instrument. Each individual session has the same fixed structure (described in Fig. 2) starting with percussion exercises as a warm-up and as a form of gross and fine mobility training (20 min), followed by keyboard exercises to train fine movements (20 min), an evaluation of the motor performance and finally, play musical games (15 min). Mood is evaluated at the end of each session (5 min).

**Table 1:** Manual for therapists enriched Music-Supported Therapy: Instruments and movements. This table provides information about the different movements that can be trained with each instrument. For sitting exercises the patient must be seated in a chair without armrests in a comfortable position with both knees and hip at 90°. For standing exercises the patient must be standing up having the chair at their back and with feet slightly separated

Instrument	Type of movement	Body position	Movement description
**Tambourine with beater**	**Shoulder flexion/extension** (shoulder at 90° and elbow at 0°)	Sitting/Standing	**Position:** Hold the tambourine with the unaffected extremity and the beater with the affected extremity. Both shoulders should be at 90° with the elbows at 0°. **Movement:** Hit the tambourine with the beater trying to keep the elbow at 0° to produce movements of shoulder flexion and extension.
	**Shoulder internal/external rotation and elbow flexion/extension** (elbow at 90°)	Sitting/Standing	**Position:** Hold the tambourine with the unaffected extremity and the beater with the affected extremity. **Movement:** Hit the tambourine with the beater to produce movements of elbow flexion and extension.
	**Wrist flexion/extension** (shoulder at 0° and elbow at 90°)	Sitting/Standing	**Position:** Hold the tambourine with the unaffected extremity and the beater with the affected extremity. **Movement:** Hit the tambourine with the beater trying to keep the elbow at 90° to produce movements of wrist flexion and extension.
**Tambourine**	**Shoulder abduction/adduction** (elbow at 0°)	Standing	**Position:** Hold the tambourine with the affected extremity. Standing up, extremities should drop on either side of the body. **Movement:** Hit the tambourine in the side of your leg trying to keep the elbow at 0° to produce movements of shoulder abduction/adduction.
	**Shoulder abduction/adduction** (elbow at 0°)	Standing	**Position:** Hold the tambourine with the affected extremity. Standing up, extremities should drop on either side of the boy. **Movement:** Hit the tambourine in the front of your leg trying to keep the elbow at 0° to produce movements of shoulder abduction/adduction.
	**Wrist flexion/extension** (shoulder at 0° and elbow at 0°)	Standing	**Position:** Hold the tambourine with the affected extremity. Standing up, extremities should drop on either side of the body. **Movement:** Hit the tambourine in the side of your leg trying to keep the elbow at 0° to produce movements of wrist flexion/extension.
	**Forearm supination/pronation** (shoulder at 0° and elbow at 90°)	Sitting/Standing	**Position:** Hold the tambourine with the affected extremity. The shoulder should be at 0° and elbow at 90°. **Movement:** Turn your forearm up and down trying to keep the elbow at 90°.
	**Forearm supination/pronation** (shoulder at 90° and elbow at 0°)	Sitting/Standing	**Position:** Hold the tambourine with the affected extremity. Hold the tambourine with the affected extremity. The shoulder should be at 90° and elbow at 0°. **Movement:** Turn your forearm up and down trying to keep the elbow at 0°.
**Maracas**	**Elbow flexion/extension** (shoulder at 0°)	Sitting/Standing	**Position:** Hold a maraca with the affected extremity. **Movement:** Starting with the elbow at 90°, shake the maraca to produce movements of elbow flexion and extension.
	**Wrist flexion/extension** (shoulder at 0° and elbow at 90°)	Sitting/Standing	**Position:** Hold a maraca with the affected extremity. **Movement:** Shake the maraca to produce movements of wrist flexion and extension trying to keep the elbow at 90°.
**Güiro**	**Elbow flexion/extension** (no gravity)	Sitting	**Position:** Hold the güiro with the unaffected extremity and the stick with the affected extremity. The güiro can rest over the knees and both elbows should be at 0° of flexion. **Movement:** Rub the sick along the notches of the güiro.
**Rainstick**	**Forearm supination/pronation** (shoulder at 90° and elbow at 0°)	Sitting/Standing	**Position:** Hold the rainstick with the affected extremity. The shoulder should be at 90° with the elbow at 0°. **Movement:** Turn your forearm up and down.
**Egg Shaker**	**Elbow flexion/extension** (shoulder at 0°)	Sitting/Standing	**Position:** Hold the egg shaker with the affected extremity. The shoulder should be at 0° and the elbow at 90°. **Movement:** Starting with the elbow at 90°, shake the egg to produce movements of elbow flexion and extension.
	**Elbow flexion/extension** (no gravity)	Sitting/Standing	**Position:** Hold the egg shaker with the affected extremity. **Movement:** Shake the egg side to side.
	**Wrist flexion/extension** (shoulder at 0° and elbow at 90°)	Sitting/Standing	**Position:** Hold the egg shaker with the affected extremity. The shoulder should be at 0° and the elbow at 90°. **Movement:** Shake the egg up and down trying to keep the elbow at 90° to produce movements of wrist flexion and extension.
**Castanets**	**Fingers mass flexion/extension** (shoulder at 0° and elbow at 90°)	Sitting	**Position:** Hold a castanet with the affected extremity, leaving the castanet resting on the palm. The elbow should be at 90° of flexion and shoulder at 0° of flexion. **Movement:** Press the castanet with the fingers to produce movements of finger mass flexion and extension.
**Djembe**	**Wrist flexion/extension** (shoulder at 0° and elbow at 90°)	Sitting	**Position:** Hold the djembe with the nonaffected extremity and lean it between your knees. The shoulder should be at 0° and the elbow at 90°. **Movement:** Hit the djembe with the affected hand trying to keep the elbow at 90° to produce movements of wrist flexion and extension.
	**Elbow flexion/extension** (shoulder at 0° and elbow at 90°)	Sitting	**Position:** Hold the djembe with the nonaffected extremity and lean it between your knees. The shoulder should be at 0° and the elbow at 90°. **Movement:** Hit the djembe with the affected hand trying to shoulder at 0° to produce movements of elbow flexion and extension.

##### Percussion exercises

**Fig. 3:**
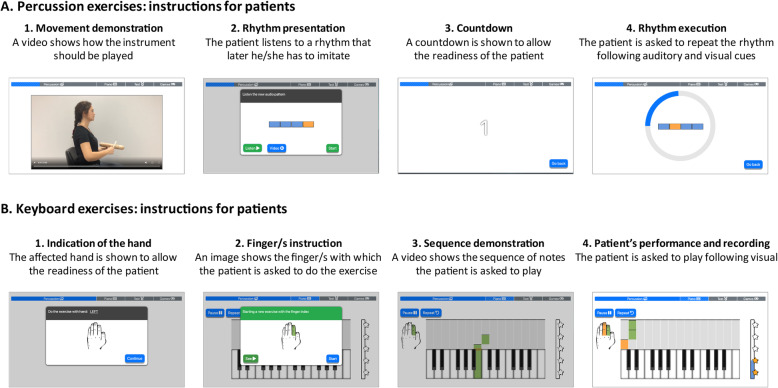
Percussion and keyboard exercises for the home-based individual sessions. **a** Percussion exercises: instructions for patients. First, there is a video to instruct how the instrument should be played (1) followed by the presentation of the rhythmic pattern (2). After a visual countdown (3), the patient has to reproduce the rhythm with the instrument (4). **b** Piano exercises: instructions for patients. A drawing of the affected hand is shown (1) followed by a cue that indicates the finger/s that should be used in the exercise (2). After a short clip showing the sequence of notes to be played (3) the patient has to play the keyboard following visual cues (4)

In each session, the participant is presented with an image of four different percussion instruments and can select the order in which she / he would like to play them. For each exercise, the app first displays a short video demonstrating how to play the particular instrument and provides instructions to avoid compensatory movements (Fig. 3a). Then, the participant is instructed to play the instrument following a rhythmic pattern as shown on the tablet. The patient is asked to listen to the rhythmic pattern for later imitation. A visual cue provides a countdown to ensure the readiness of the patient. Then, the patient is asked to reproduce the pattern following auditory and visual cues. Table 2 describes the rhythmic patterns of the percussion exercises, which are graded by difficulty. The different movements, rhythmic patterns and tempo involved in the percussion exercises are presented in Table 3.

**Table 2:** Rhythmic patterns. This table provides the different rhythmic patterns that are asked to play in the home-based MST sessions with different percussion instruments

Rhythmic patterns	
**Level 1**	
**Level 2**	
**Level 3**	
**Level 4**	
**Level 5**	
**Level 6**	
**Level 7**	
**Level 8**	

##### Keyboard exercises

For the exercises with the keyboard, participants are asked to play simple sequences that gradually increase in difficulty (Fig. 3b). The app provides visual instructions as well as cues to prompt finger movements and records the performance of the patient with the keyboard. Exercises with the keyboard increase in difficulty depending on the patient’s progress. During each exercise, different types of feedback encourage the participant to play as well as they can. A vertical score bar on the right side of the screen with a star on the top fills up as the participant plays the notes correctly. In addition playing an incorrect note causes the screen to turn red so that the participants can realize their mistake and play the correct note.

##### Games

Participants have the possibility to play musical games, an optional part of the treatment they can skip if they are tired or not in the mood to play. They can choose between four different games: 1) *Simon says*, comprising the participant is asked to repeat a sequence that is presented, which becomes progressively longer and more complex as she / he succeeds; 2) *Piano Hero*, notes scroll on-screen to the different keys and participants have to play in time to score points; 3) *Combo*, the participant plays along songs that are rehearsed during the group sessions by accompanying the melody with the keyboard and/or using the percussion instruments; and 4) *Impro,* the participant improvises freely on the keyboard.

##### Monitoring

The keyboard is connected to the electronic tablet as a midi-device, which allows recording the played notes, any errors made, key-pressure and the execution time for each exercise. The AI platform is based on three main components (visualisation, prediction, and prescription). All the information is processed to visually summarise the performance of the patient. To tailor the intervention to the individual needs of the patient, the therapist can virtually prescribe exercises and instruments to be used for each session. The prediction component computes an initial performance estimation based on baseline assessments. The prescription component recommends appropriate exercises to the therapist by taking into account the performance of the patient and adjusting them for difficulty. Since the percussion instruments have no sensors, the AI platform only gathers data related to the interaction with the app (watching the video, listening to the rhythm, and starting the exercise).

##### Daily evaluation

At the end of the home-based sessions the motor performance and the mood of the participants are evaluated. Five keyboard exercises of 3–5 min duration are applied ranging in difficulty from playing simple sequences with one finger to playing complex sequences with five fingers. Moreover, participants are asked to rate their mood on a visual scale of emotional faces (from *1 = I am feeling very bad* to *5 = I am feeling very good*). Participants can also record a voice message for the therapist. The therapist’s response is shown at the beginning of the following session.

#### Group sessions

Once per week a one-hour virtual group session of music therapy will be conducted using a video communication platform that is installed in the electronic tablet provided to participants. Participants will be split into groups of 3–4 people for the group sessions. A music therapist and an occupational therapist will conduct the group sessions, providing instructions for the music therapy exercises. The sessions involve active and passive music therapy exercises structured into three parts: 1) *Beginning*, featuring warming-up exercises; 2) *Core*, comprising musical improvisation and playing of favourite songs; and 3) *Ending*, featuring relaxation exercises. All the exercises will be accompanied by live music with the music therapist playing along on the piano or guitar. Participants will be asked to play musical instruments during the sessions to train gross and fine motor skills, but they can select the musical instruments they wish to play from many different percussion instruments. Participants will be encouraged to play with the affected extremity to showcase what they have learned during their individual sessions. At the beginning and the end of the session, therapists will facilitate a discussion where patients can share their progress, difficulties and worries. The selection of songs for the group sessions is done with the participants, taking into account their musical preferences. For this reason, a semi-structured interview will be conducted at the beginning of the intervention program to understand cultural preferences, professional and leisure activities, musical knowledge and previous musical experience and preferences.

**Table 3:** Percussion exercises. For each percussion exercise, the movements involved, and the rhytmic pattern and tempo that can be asked to the participant is presented

Instrument	Movement	Rhythmic patterns	Tempo
**Tambourine with beater**	**Shoulder flexion/extension** (shoulder at 90° and elbow at 0°)	1, 2, 3	30
	**Shoulder internal/external rotation and elbow flexion/extension** (elbow at 90°)	1, 2, 3, 4, 7, 8	40
	**Wrist flexion/extension** (shoulder at 0° and elbow at 0°)	1, 2, 3, 4, 5, 6, 7, 8	60
**Tambourine**	**Shoulder abduction/adduction** (elbow at 0°)	1, 2, 3	30
	**Shoulder abduction/adduction** (elbow at 0°)	1, 2, 3	30
	**Wrist flexion/extension** (shoulder at 0° and elbow at 0°)	1, 2, 3, 4, 5, 6, 7, 8	60
	**Forearm supination/pronation** (shoulder at 0° and elbow at 90°)	1, 2, 3, 4, 5, 6, 7, 8	60
	**Forearm supination/pronation** (shoulder at 90° and elbow at 0°)	1, 2, 3, 4, 5, 6, 7, 8	60
**Maracas**	**Elbow flexion/extension** (shoulder at 0°)	1, 2, 3, 4, 6, 8	50
	**Wrist flexion/extension** (shoulder at 0° and elbow at 90°)	1, 2, 3, 4, 5, 6, 7, 8	60
**Güiro**	**Elbow flexion/extension** (no gravity)	1, 2, 3, 4, 5, 6, 7, 8	60
Rainstick*	**Forearm supination/pronation** (shoulder at 90° and elbow at 0°)	**–**	**–**
**Egg Shaker**	**Elbow flexion/extension** (shoulder at 0°)	1, 2, 3, 4, 6, 8	50
	**Elbow flexion/extension** (no gravity)	1, 2, 3, 4, 5, 6, 7, 8	60
	**Wrist flexion/extension** (shoulder at 0° and elbow at 90°)	1, 2, 3, 4, 5, 6, 7, 8	60
**Castanets**	**Fingers mass flexion/extension** (shoulder at 0° and elbow at 90°)	1, 2, 3, 4, 5, 7, 8	60, 70
**Djembe**	**Wrist flexion/extension** (shoulder at 0° and elbow at 90°)	1, 2, 3, 4, 5, 6, 7, 8	60, 70
	**Elbow flexion/extension** (shoulder at 0° and elbow at 90°)	1, 2, 3, 4, 5, 6, 7, 8	50

##### Daily evaluation

At the end of the group session, each participant will be asked to answer the *Participant Post-Session Questionnaire*, consisting of 4 short questions about the feelings of connection to music and the people in the group on a 4-point Likert scale. Moreover, the therapists evaluate the overall performance of each patient as well as their engagement in music making using the 9-point *Music Engagement Scale* adapted from the *Music Therapy Communication and Social Interaction scale* (MTCSI; [13]). Each participant is also asked to rate the social connectedness with others following the *Inclusion of Other in the Self Scale* [6], and participants have to rate their mood using a visual scale of emotional faces (from *1 = I am feeling very bad* to *5 = I am feeling very good*).

### Control treatment: GRASP

Participants in the control intervention group will follow the Graded Repetitive Arm Supplementary Program (GRASP, [36]). This program consists of self-directed arm and hand exercises for stroke patients and it is validated to be performed by patients on their own at home. GRASP seeks to promote hand and arm motor function recovery through mass repetition of movements and task-specific exercises, encouraging the use of the affected extremity in everyday activities. Participants will be asked to complete 4 weekly one-hour session for 10 weeks (total program duration: 40 h).

A booklet describing the exercises and the equipment needed will be provided to participants. Exercises involve arm and hand strengthening, coordination and manual skills using everyday objects (e.g., toothpicks, Lego bricks, clothespins or paper clips). The participant will be asked to perform the exercises sitting in a chair or next to a table in sets of 5 or 10 repetitions. The number of sets and the exercises prescription will be adjusted to the patient’s needs and endurance.

At the beginning of the intervention, the therapist will visit the participant’s home to provide instructions and equipment to follow the program. Patients will be asked to register the type and amount of exercises for each session on a record sheet. Moreover, after each session, participants will be asked to rate their mood using a visual scale of emotional faces (from *1 = I am feeling very bad* to *5 = I am feeling very good*). The therapist will contact the participant by phone once a week to monitor her/his progress and difficulties.

### Treatment compliance and withdrawal

Completion of intervention protocol would require the patient to perform ≥80% of the sessions.

### Evaluation

An initial evaluation will include the collection of demographical and clinical variables will be performed at baseline. In addition, primary and secondary motor, cognitive and emotional well-being and quality of life outcomes will be evaluated before and after the intervention as well as at 3-month follow-up. Table 4 summarises the variables and outcomes of the study. Considering the current health crisis due to the COVID-19 pandemic, the evaluation will be carried out at the participant’s home in order to avoid unnecessary visits to the hospital.

**Table 4:** Variables and outcomes of the study

Instrument	Reference	Baseline	Post-intervention	Follow-up
**Demographic and clinical variables**				
Montreal Cognitive Assessment	[62]	•		
Digit span subtest	[97]	•		
Boston Naming Test, short form	[47]	•		
Vocabulary subtest	[97]	•		
Scale and rhythm subtests Montreal Battery of Evaluation of Amusia	[68]	•		
Barcelona Music Reward Questionnaire	[51]	•		
Grit scale	[23]	•		
Multidimensional Scale of Perceived Social Support	[95]	•		
**Primary outcome**				
Action Research Arm Test	[46]	•	•	•
**Secondary motor outcomes**				
Fugl-Meyer Assessment of Motor Recovery after Stroke	[27]	•	•	•
Grip strength	[54]	•	•	•
Box and Block Test	[53]	•	•	•
Nine Hole Pegboard Test	[66]	•	•	•
Chedoke Arm and Hand Activity Inventory	[7]	•	•	•
**Secondary cognitive outcomes**		•	•	•
Behaviour Rating Inventory of Executive Function	[74]	•	•	•
Sustained Attention to Response Task	[72]	•	•	•
Figural memory subtest	[90]	•	•	•
Rey Auditory Verbal Learning Test	[74]	•	•	•
Fluency test	[89]	•	•	•
**Secondary emotional well-being and quality of life outcomes**				
Beck Depression Inventory-II	[11]	•	•	•
Apathy Evaluation Scale	[49]	•	•	•
Profile of Mood States	[57]	•	•	•
Stroke Impact Scale	[61]	•	•	•
**Self-regulation and self-efficacy outcomes**		During intervention		
Treatment Self-regulation questionnaire	[80]	•		
Treatment Questionnaire Concerning Continued Program	[80]	•		
Intrinsic Motivation Inventory	[79]	•		
Strategies Used to Promote Health Questionnaire	[46]	•		

#### Baseline demographic and clinical variables

Demographic and clinical variables such as age, gender, level of education, living situation, previous musical training, stroke aetiology and location, and lesion laterality will be collected from medical records as well as during a first interview with the participant.

In order to characterise individual differences that can have a mediating effect on treatment success, we will evaluate cognitive functions, differences in the processing and integration of music, music reward, perseverance and social support at the beginning of the intervention. Therefore, the baseline evaluation will include the Spanish version of the *Montreal Cognitive Assessment* [45, 62] to assess global cognitive function, the *digit span* (included in the *Wechsler Adult Intelligence Scale,* WAIS-IV, [97]; adapted to Spanish by Pearson Clinical & Talent Assessment) to assess working memory, a short version of the *Boston Naming Test* validated in Spanish [14, 47] to assess language production, and the *vocabulary subtest* of the WAIS-IV. In addition, the *scale* and *rhythm subtests* from the shortened version of the *Montreal Battery of Evaluation of Amusia* [68] will be used to screen for amusia at baseline. Participants will complete the *Barcelona Music Reward Questionnaire* ([51], validated in Spanish) on the first evaluation to assess individual differences in pleasure derived from musical experiences. Finally, individual differences in perseverance and passion for long-term goals will be assessed with the *Grit Scale* ([23]; validated in Spanish in [9]), and social support will be assessed using the Spanish version of the *Multidimensional Scale of Perceived Social Support* ([43, 95]).

#### Primary outcome

The primary outcome will be upper extremity function measured with the *Action Research Arm Test* (ARAT, [46]). The ARAT is recommended for use in chronic stroke and outpatient rehabilitation by the StrokeEDGE Task Force Group [81] and has excellent test-retest and inter/intra-rater reliability [69, 85]. The measure is a 19-item test divided into four subtests (grasp, grip, pinch and gross movement). For each item, the patient is asked to perform a simple task that involves a functional movement of the affected upper limb. Each task is rated using a 4-point ordinal scale. The maximum possible score is 57 and the minimal clinically important difference is 5.7 points [84].

#### Secondary outcomes

##### Motor outcomes

The *upper extremity subtest* of the *Fugl-Meyer Assessment of Motor Recovery after Stroke* (FMA [27];) will be used to evaluate motor impairment; grip strength will be assessed with a dynamometer and functional movements and dexterity will be assessed with the *Box and Block Test* [53] and the *Nine Hole Pegboard Test* [66]. The *Chedoke Arm and Hand Activity Inventory* will be used to evaluate the patients’ motor performance in everyday tasks [7].

The upper extremity subtest of the *FMA* comprises 33 items that evaluate motor impairment in the affected upper limb. The test is divided into 4 sections (shoulder, forearm and elbow, wrist, hand and coordination) assessing reflexes, flexor and extensor synergies, range of motion, and overall coordination and speed of the upper extremity. Each item is graded using an ordinal scale from 0 to 2. The maximum possible score is 66 and the minimal clinically important difference for chronic stroke patients is 5.2 points [64].

The grip strength will be measured for both hands as the mean of three consecutive trials [54].

The *Box and Block Test* assesses gross manual dexterity by using a setup consisting of a box with 2 compartments and wood cubes. The participant is asked to grasp cubes and transport them from one compartment to the other. The number of cubes successfully transported within 1 minute is scored for the affected and the unaffected extremity. The minimal detectable change for the Box and Block Test is 5.5 cubes (H. M [16].).

The *Nine Hole Pegboard Test* evaluates fine dexterity by asking the patient to pick up nine pegs and place them into holes on a board and then remove them. The patient is asked to complete this task as fast as possible and the time needed is scored for the affected and the unaffected hand. The minimal detectable change for this test is 32.8 s (H. M [16].).

The *Chedoke Arm and Hand Activity Inventory* is a performance test that measures the patient’s ability to perform everyday tasks with both extremities. The test is composed of 13 different tasks (i.e. open a jar of coffee, make a phone call, clean a pair of eyeglasses) and each task is graded using an ordinal scale from 1 to 7. The scores are given based on the involvement of the affected extremity in the task, ranging from less than 25% of involvement in the task needing total assistance to complete independence in the task (adequate time and safety). The minimal clinically importance difference is 6.3 points [8].

##### Cognitive outcomes

The neuropsychological evaluation will focus on assessing executive function, attention, visuospatial memory and verbal learning, and fluency. Executive function will be evaluated using the *Behaviour Rating Inventory of Executive Function* (BRIEF, [74]; validated in Spanish in [10])*.* The *Sustained Attention to Response Task* [72] will be used to assess sustained attention whereas the *Figural Memory subtest from the Wechsler Memory Scale-Revised* ([90]; adapted to Spanish by Pearson Clinical & Talent Assessment) will evaluate visuospatial memory. Verbal learning will be evaluated using the Spanish version of the *Rey Auditory Verbal Learning Test* [50] and a *fluency test* in Spanish [67] will be used to examine verbal fluency.

The adult version of the *BRIEF* is a self-report questionnaire that evaluates executive functioning in everyday life situations. The questionnaire includes a self and informant reports. Both versions comprise 75 items describing various behaviours, and the participant is asked to report if the behaviour is never a problem, sometimes a problem or often a problem. The BRIEF provides two broad indexes (behavioral regulation and metacognition) as well as an overall score. It includes nine scales that assess the ability to inhibit, self-monitor, plan/organize, shift, initiate, task monitor, emotional control, working memory, and organization of materials.

The *Sustained Attention to Response Task* is a computerised test that evaluates sustained attention by presenting the participant with one number at a time and asking him or her to respond as fast as possible when a target number appears. This test provides measures of reaction times and changes on this variable over the task, as well as inhibition errors.

The *Figural Memory subtest from the Wechsler Memory Scale-Revised* [90] measures visuospatial recall and recognition memory. The participant is presented with abstracts designs that later he or she has to identify from an array.

The *Rey Auditory Verbal Learning Test* evaluates verbal learning by asking the patient to recall a list of 15 words. The list is presented 5 times and after each trial, the patient is asked to repeat as many words as he/she recalls. Then, a second list of words is presented as interference. The number of words recalled from the original list after the interference and after a break of 20 min is determined. A recognition test with distractors is also performed at the end of the test.

The *verbal fluency test* comprises two tasks: category and letter fluency. The participant is asked to produce as many words as possible for a minute of the same semantic category (i.e. animals) or words that start with the same letter (i.e. words starting with ‘p’). The number of unique words produced is counted as the score for each task.

##### Emotional well-being and quality of life outcomes

The emotional well-being evaluation will assess depression with the Spanish version of the *Beck Depression Inventory-II* [11, 76], apathy with the *Apathy Evaluation Scale* translated to Spanish [49] and mood with the *Profile of Mood States* ([57]; Spanish version by [5]). Health-related quality of life will be measured with the *Stroke Impact Scale,* which has been translated to Spanish for this study [61].

The *Beck Depression Inventory-II* is a self-report measure that comprises 21 multiple-choice questions that are scored on a scale from 0 to 3. The participant is asked about feelings, thoughts and behaviours of the past week. Higher scores indicate depression severity and the maximum possible score of the measure is 63.

The *Apathy Evaluation Scale* evaluates behavioural, cognitive and emotional indicators of apathy. The scale comprises a self and informant reports both consisting of 18 items that are scored on a 4-point Likert scale, where higher scores indicate more apathy.

The *Profile of Mood States* evaluates different dimensions of mood by asking the participant to rate feelings or emotion felt during the past week. The measure includes 65 items that are scored on a 5-point Likert scale ranging from 0 “*not at all*” to 4 “*extremely*”.

The *Stroke Impact Scale* is a 59-item self-report questionnaire that assesses muscle strength, hand function, basic and instrumental activities of daily living, global mobility, communication, emotion, memory and thinking, and participation.

##### Self-regulation and self-efficacy outcomes

Domain-specific individual differences in motivation and self-regulation for joining, participating in and engagement during the rehabilitation program will be assessed with the *Treatment Self-Regulation Questionnaire* (adapted from: [80]), the *Treatment Questionnaire Concerning Continued Program Participation* (adapted from: [80]), and the *Intrinsic Motivation Inventory* [79], respectively. Self-efficacy will be measured with the *Strategies Used by People to Promote Health* questionnaire [46]. These questionnaires have been translated to Spanish for the purpose of the study.

The *Treatment Self-Regulation Questionnaire* is an adaptation of the standard version called *Academic Self-Regulation Questionnaire (SRQ-A)* [80] created for the study. It evaluates the type of self-regulation (external, interjected, identified or intrinsic) or motivation (external or intrinsic) of the participants to engage with the rehabilitation program. It is a 15-item self-report questionnaire scored on a 7-point scale (*1: not all true; 4: somewhat true; 7: very true*). The items are questions about the reasons why the participants enrolled in the current study. Thus, the participants will be asked to answer it only at the beginning of the intervention.

The *Treatment Questionnaire Concerning Continued Program Participation* evaluates the type of self-regulation or motivation of the participants for continuing to participate in the rehabilitation program to which they have been assigned. It is a 15-item self-report questionnaire scored on a 7-point scale (*1: not all true; 4: somewhat true; 7: very true*). The items are questions about why the participants continue to engage with the rehabilitation program. Hence, the participants will be asked to complete it only in the middle of the intervention (the 5th week).

The *Intrinsic Motivation Inventory* is a multidimensional measure which assesses the content and level of motivation during an intervention. Therefore, participants will be asked to answer it only on completion of the rehabilitation program. The measure was adapted for the current study and consists of 24 self-report questions divided into six different psychological constructs reflecting positive or negative predictors of intrinsic motivation: 1) interest/enjoyment; 2) perceived competence; 3) effort/importance; 4) pressure/tension; 5) perceived choice; and 6) value/usefulness. It is scored on a 7-point scale (*1: not all true; 4: somewhat true; 7: very true*). The MST-group will complete 4 additional items on the psychological construct of relatedness in order to evaluate the effect of the group sessions on motivation levels.

The *Strategies Used by People to Promote Health* is a 29-item self-report questionnaire that evaluates the degree of self-care and self-efficacy through four factors consistent with the underlying self-efficacy theory upon which the scale is based: 1) coping, 2) stress reduction, 3) making decisions, and 4) enjoying life. It is scored on a 5-point scale (from *1: very little confidence* to *5: quite a lot of confidence*) and the participants will be asked to answer it in both pre- and post-intervention evaluations.

### Blinding

A clinical researcher with expertise in stroke rehabilitation will perform the evaluations and will be blinded to participants’ group allocation.

### Randomisation

Participants will be randomised to one of the groups following a block randomisation procedure. As the eMST program requires three participants for the group sessions, randomisation will consider clusters of three participants. The randomisation sequence will be computer-generated and only accessible to a research assistant who will not be involved in the recruitment, treatment or evaluation of participants. This research assistant will inform the therapists once the baseline evaluation of the participant is done.

### Ethical considerations and data management

The protocol of this study has been approved by the Clinical Research Ethics Committee of the Bellvitge University Hospital (PR095/17; Barcelona, Spain) and the Hospitals del Mar i l’Esperança (Parc de Salut Mar, 2020/9523; Barcelona, Spain) and follows the Declaration of Helsinki to experiment with human beings. As mentioned previously, participants will be informed about study procedures and will sign an informed consent form prior to participation. Each participant will be identified with a code when collecting demographic and clinical variables and outcomes. All the information gathered by the app will be stored on an internal secure server from the Artificial Intelligence Research Institute, which is maintained by the IT team following institutional security standards. Additionally, patients are de-identified using the generated code. Connections between the tablet and server use a secure credential in an API Rest through a HTTPS protocol.

### Sample size

The sample size calculation is based on a clinically relevant difference between treatment groups with 80% power and a 5% significance level. Considering that the minimal detectable change of the ARAT (primary outcome) is 5.7 points and that an acceptable difference between groups of 15% is defined to be clinically meaningful [84, 85], a sample size of 26 participants will be required in each group. From experience gathered in previous studies [32, 77], the dropout rate in this type of interventions is relatively low (15%). Taking this rate into consideration, a final estimation of 60 participants is needed in the study, 30 per group.

### Data analysis

Statistical analysis will be performed using *R* (R Core Team, 2019) and the *Statistical Package for the Social Sciences* (SPSS version 21, SPSS inc., Chicago, Illinois, USA). For descriptive analyses, quantitative data will be analysed using mean and standard deviation if the data is normally distributed and median and interquartile range will be used for variables that are not normally distributed. Qualitative variables will be presented using frequency distributions and percentages.

An intention-to-treat analysis will be performed for all outcome variables including all patients that were assigned to a group and for whom the primary outcome was collected at baseline. In the case of participants that withdraw form the study, the score at baseline will be assigned for all the evaluation points and reasons for withdrawal will be reported.

The effect of the intervention on the primary outcome, the ARAT, will be assessed using the chi-square test. Taking into account the minimal clinically important difference of this test (5.7 points) data will be dichotomized into two categories: *clinically improved* or *unchanged/deteriorated.* An analysis of covariance (ANCOVA) will be carried out for all secondary outcomes to determine differences between groups across time using demographical and clinical baseline variables as covariates. The level of significance will be set at *p* < 0.05 for all statistical tests and corrected for multiple comparisons when necessary.

### Trial registration

The trial has been registered at ClinicalTrials.gov and identified as NCT04507542.

## Discussion

Chronic stroke patients with residual motor deficits experience limitations in activities of daily living and restrictions in participation in community life [35]. The impact of these residual motor deficits on function coupled with low levels of physical activity can lead to poor emotional well-being, life satisfaction and quality of life [12, 38]. There is increasing interest in developing home and community-based interventions for chronic stroke patients to address their needs and improve their autonomy, participation and overall well-being [13, 34].

MST aims at improving motor and cognitive functions, emotional well-being and quality of life in stroke patients and could be a feasible intervention to apply in the chronic phase [32, 71, 79]. We have adapted the original MST program for home use and we have increased training intensity and variability, incorporated group sessions, and modified the protocol to boost learning by enhancing motivation and promoting more autonomy. The incorporation of these changes and the adaptation of the program take into account the Medical Research Council Framework for Developing and Evaluating Complex Interventions. In order to further develop MST we identified relevant evidence in the literature and developed a theoretical understanding of the processes of change [33]. Moreover, we have modelled the intervention process and outcomes, and assessed feasibility with a pilot study.

The study has been designed following the CONSORT guidelines for conducting clinical trials. The evaluation protocol includes baseline variables such as social support, perseverance or musical reward to take into account individual differences that may modulate the treatment effect. The outcomes of the study address various dimensions of functioning, with instruments that evaluate body functions, activity and participation. In addition, we have included measures of self-regulation and self-efficacy. Considering that the intervention is home and community-based, these factors may have a crucial role in treatment adherence and success.

We expect that patients treated with eMST will show larger functional improvements than patients treated with the control therapy regimen. On the basis that MST improves several dimensions of functionality beyond the motor function, we expect that patients will improve their mood and emotional well-being after MST.

## Data Availability

Data and materials can be accessed by contacting the corresponding author and in the research group webpage: https://www.brainvitge.org

## Abbreviations

AI: Artificial intelligence
ARAT: Action Research Arm Test
BRIEF: Behaviour Rating Inventory of Executive Function
eMST: Enriched Music-Supported Therapy
FMA: Fugl-Meyer Assessment of Motor Recovery after Stroke
GRASP: Graded Repetitive Arm Supplementary Program
MST: Music-Supported Therapy
MTCSI: Music Therapy Communication and Social Interaction scale
